# Supplementary material for: Non-Cooperation within a School-Based Wellness Program during the COVID-19 Pandemic—A Qualitative Research
Source: Int J Environ Res Public Health. 2022 Jun 2;19(11):6798. doi: 10.3390/ijerph19116798 (PMC9180888; doi:10.3390/ijerph19116798)
Supplement: Supplementary file 1 [file ijerph-19-06798-s001.zip › ijerph-1710223-supplementary.pdf]

Supplementary Material S1

Table S1. Topics and description of program sessions

1.	Introduction to "Favoring My Self"	Acquaintances; Discussion of expectations; Introduction of the program and its goals; Establishment of a group contract.
2.	Self-care moderation	Importance of self-care. Discussing the recommendations and practices of sleeping and eating hygiene. Sharing self-care experiences and outcomes. Understanding the principle of balance and moderation vs. extremes/perfectionism.
3.	Self-preservation	Discussing territorial issues and self-space with of examples from the animal world and participants' experiences, as well as discussing identification and management of media violence.
4.	Media literacy	Identification of the hidden media messages in relation to media messages; Learn about advertisement strategies used by the media; Development of critical thinking regarding these messages.
5.	Our feelings	Facilitating a "feeling differentiation" activity. Sharing observed and hidden feelings. Suggesting management strategies and practicing emotional conversation.
6.	Accepting appearance differences	Discussing current societal ideals of appearance, considering their unrealistic and narrow construction. Practicing strategies to avoid and challenge comparisons.
7.	Accepting our weaknesses	Distinguishing between primary image and authentic self. Examples of people who turned their defects into productive affects. Accepting disadvantages or weaknesses that cannot be changed.
8.	My body and I	Recognition of adolescence as a period of various changes that are often uncontrollable; Role play scenario with strategies of managing and accepting these changes.
9.	Adolescent rights and responsibilities	Promoting the desire to grow up due to the extensive opportunities it provides and despite its inherent responsibilities.
10.	Summary and commitment	Reviewing key messages. Committing to engaging in positive self-care and positive body-image behaviors and rejecting risk factors.

Supplementary Material S2

Thematic semi-structured interview guide: Non-Cooperation Within a School-Based Wellness Program During the COVID-19 Pandemic - A Qualitative Research

Opening question: Tell me about your experience in "Favoring Myself"

Additional probs were used to ensure that all topics were covered. Each interview was different; however, these were the typical probs:

- What was your general impression of the program?
- Would you please explain this perception in the context of your aims?
- What did you gain personally from the delivery of the program to your children?
- What were you uncomfortable or angry about?
- How did you decide not to participate in the program? What did you explain to your child regarding your choice?
- What barriers did you have to face to collaborate with the program assignments?
- What would you suggest doing differently next year?
- What did you like best in the program?
- Would you suggest that your friends participate in it next year?
